# Case in Which the External Iliac Artery Was Tied under Peculiar Circumstances

**Published:** 1827-08

**Authors:** H. L. Gibbs

**Affiliations:** the General Naval Hospital, St. Petersburgh.


### HEMORRHAGE.

*Case in which the external Iliac Artery was tied under peculiar Circumstances, by* H. L. GIBBS, M.D. at the GENERAL NAVAL HOSPITAL, St. Petersburgh.

ALEXIS KATCHALOF, æt. thirty-nine, a seaman, of robust make, light hair, blue eyes, and of a scrofulous complexion, was admitted into the General Naval Hospital of St. Petersburgh, on the 14th April, 1826, with a small superficial ulcer on the pubis, and induration of the mass of femoral and inguinal glands of the left thigh. As this was supposed to be syphilitic, he was placed in the venereal wards, though fortunately, perhaps, not treated with mercury. The ulcer soon healed; but abscesses formed from time to time, leaving extensive infiltrations below the groin. The sinuses were occasionally laid open, and phagedenic ulceration supervened. Thus more than nine months passed away, the health of the patient gradually declining. In the early part of December, the fascia of the groin and upper part of the thigh began to slough, together with the remainder of the glands. Under the assiduous care of Dr. GORONINOF, this was subdued; bark, wine, and mineral acids, being administered; and the Liquor Choloreti Calcis, in the proportion of one part to five ounces of water, repeatedly and effectually used as a lotion.

By the middle of January, 1827, granulation had commenced throughout the whole of this extensive sore; a small space at the centre, over the great vessels, excepted.

Feb. 1st.—Hemorrhagy from the femoral artery took place, but was speedily restrained by the application of the finger. My assistance was required, and, happening to be at the hospital at the time, I soon secured the bleeding vessel a hand's-breadth below Poupart's ligament, by two ligatures; the one an inch above, the other as much below, the ulcerated aperture, having merely the granulations to divide for this purpose. I cut the artery through between the ligatures. The vessel at the parts tied had no morbid appearance.

The patient now fell under my care, and was placed in a separate room near the operation theatre. He was in a state of extreme emaciation, with hectic fever commencing. The Inf. Rosar. acid. with a moderate quantity of port-wine, mixed with lemonade, was prescribed. The limb was covered with flannel; a long narrow sack or bag of heated bran placed on each side; and a stone bottle of warm water to the sole of the foot.

9th.—The limb warm; applications continued. Very slight œdema of the leg and foot. Appetite and general health improved. At the evening dressing, the lower ligature came away.

Feb. 10th.—The upper ligature was thrown off, but, as before, the hemorrhagy was soon suppressed by the attendant. During my absence, the artery was tied an inch and a half higher up by Dr. Goroninof. Above this part the groin was occupied by a callous cicatrix extending across it. From this period to the 25th, all went on to my satisfaction; the limb in a favourable state, and febrile exacerbations less frequent.

26th.—Early on this day, whilst endeavouring to turn himself a little in bed, a saltus took place; but the assistant, being constantly on the watch, commanded the bleeding by pressure on the inguinal artery. It was supposed that from eight to ten ounces of blood had, on these various occasions, escaped. I lost no time in rendering assistance, and, aided by Dr. Bouialski, and in presence of many of the medical gentlemen of the hospital, proceeded to take up the *external iliac artery*. As indurated glands were to be traced for some distance above Poupart's ligament, I made the incision much higher up than is usual in operating for aneurism in the groin. By means of a long and well-curved instrument, a strong round ligature was passed under the artery, and tied just below the division of the common trunk into the two iliacs. The wound was brought into contact by straps of adhesive plaster, supported by compresses and bandage. The symptomatic fever, subsequent to the operation, was of short duration, and seemed to have the good effect of rousing the system to fresh energy; for, on the 5th of March, an evident amendment and healthy action were obvious. A more generous diet, with wine and small doses of the Sulphat of Quinine, ordered. The limb, which had been previously prepared, as it were, for this operation, did not manifest any change of temperature.

March 8th, (*thirteenth day from the operation.*)—The ligature came away with the dressings. The suppuration after this became more abundant, and required the use of graduated compresses.

23d.—Health and spirits considerably improved. Limb regaining its strength and plumpness. The natural warmth appears to be returned, and he begins to move the joints.

April 2d.—Cicatrisation had extended nearly over the whole of the sore on the thigh, when an attack of erysipelas came on, and the newly-formed cuticle gave way. A dose of Calomel, followed up by gentle laxatives, removed the gastric affection which had caused this.

9th.—The patient again rallied, and was allowed to return gradually to a nourishing diet. Decoct. Cinchonæ Peruv. cum Elix. Sulph. aromat. now employed. Wound healed at the lower part: an infiltration, however, which had taken place between the integument and the external oblique muscle, required the use of the bistoury.

20th.—The sinus filling up with healthy granulations.

30th.—Wound of the abdomen healed; that below the groin returned to a healthy state, and considerably reduced in size.

May 14th.—The limb of the natural heat and appearance, and, were it not for the remainder of the sore and extensive cicatrix on the upper part of the thigh, the patient would be enabled to walk about freely.

This case may be considered interesting both in a physiological and pathological point of view, as it not only forcibly attests the wonderful power of nature in restoring circulation to an extremity, and that too under the influence of disease, but also of causing the return of lymph to the body, after the destruction of the femoral and inguinal glands. That absorption is well carried on, is evinced by the want of œdema of the limb. How this takes place I leave to abler judges than myself to decide: whether by anastomoses of the deeper-seated lymphatics, (assisted by those extending along the buttock,) or by venous absorption, the theory of which may appear to some to be hereby corroborated.

*St. Petersburgh; 15th May,* 1827.

